# Peer Mentoring Programs for Culturally and Linguistically Diverse Refugee and Migrant Women: An Integrative Review

**DOI:** 10.3390/ijerph191912845

**Published:** 2022-10-07

**Authors:** Shelley Gower, Zakia Jeemi, David Forbes, Paul Kebble, Jaya A. R. Dantas

**Affiliations:** 1 Curtin School of Nursing, Curtin University, Perth 6102, Australia; 2 Curtin School of Population Health, Curtin University, Perth 6102, Australia; 3 Faculty of Health Sciences, Curtin University, Perth 6102, Australia

**Keywords:** peer mentoring, refugee, migrant, women, host country, integration, settlement, community support programs, integrative review

## Abstract

Refugee and migrant women experience personal, cultural and structural challenges as they adapt to new lives in host countries. Peer mentoring programs are used to facilitate resettlement, build empowerment and improve job-readiness for refugee and migrant women; however, the effectiveness of these programs is not well understood. A systematic search of five databases, plus grey literature from January 2005 to December 2020, was undertaken, resulting in 12 articles. A narrative synthesis using thematic analysis identified the key components and outcomes of effective programs. Most mentoring programs were co-designed with community-based service providers, using participatory approaches to ensure cultural acceptability. Communication and sharing were facilitated using workshops and individual in-person or telephone mentoring. The training and support of mentors was critical. However, differences in expectations between mentors and mentees at times resulted in attrition. Qualitative evaluation revealed enhanced social support, greater empowerment and confidence for the women. There was improved access to the social determinants of health such as education, but limited success in obtaining employment. Mentoring programs can enhance refugee and migrant women’s wellbeing and social connectedness in resettlement contexts. However, it is unclear whether these benefits can be sustained over the longer term. Future programs should be rigorously evaluated through qualitative and quantitative analyses to generate conclusive evidence for best practice.

## 1. Introduction

There is a high and growing number of displaced people globally due to civil conflicts, war and climate impacts. This includes those who are internally displaced, and refugees and asylum seekers who are forced to leave their homelands and seek shelter in other countries due to civil conflicts, persecution and poor employment opportunities. More than 100 million people are reported to have been forcibly displaced as of June 2022, the vast majority of whom were hosted in neighboring countries, often in temporary facilities [1,2].

One durable solution available to people experiencing displacement is resettlement through the United Nations High Commissioner for Refugees (UNHCR), usually in high-income countries, which often results in permanent residency or citizenship in the new country [3]. Between 2010 and 2019, approximately 1.1 million refugees were resettled globally, and 322, 400 were naturalized in the resettlement country. Resettlement countries are often geographically distant and have a very different ethnic and national culture to the home or asylum countries of displaced people [4]. This differs from the experience of forced migration to neighboring countries, where although there may be cultural and kinship ties, and people may be integrated legally and culturally, citizenship is less likely [5,6]. 

In addition to migrants fleeing persecution, many skilled migrants make the conscious decision every year to move to other countries to take up employment opportunities and reunite with family. Combined, global migration in 2020 was 281 million with the increasing feminization of migration occurring as the number of women migrants increase [7,8]. In this paper, refugees are defined as those that have fled their home country due to the risk of serious human rights violations and persecution; and migrants are defined as those that leave their home country of their own volition for purposes related to work and employment, study, or joining family members [9]. This review focusses on peer mentoring programs for culturally and linguistically diverse (CALD) women including both refugee and migrant women that have resettled in high-income countries such as, but not limited to, Australia, New Zealand, Canada, the USA, and the UK.

### 1.1. Stress of Immigration

Migration is recognized as being a significant stressor, especially for refugees who experience substantial displacement, emotional and physical trauma, and the associated cultural dissonance of resettlement experiences, before and after migration to the host country [10,11]. Refugees may lose social capital through spending extended periods in refugee camps, and are more likely to be unemployed, have mental health concerns and experience isolation in the resettlement country [12,13]. Refugees also experience barriers to the social determinants of health such as education [14,15] and may have difficulties accessing health care [13,16,17].

Post-migration stressors such as concerns about housing, particularly shared housing, difficult interactions with government and community service providers, and limited help from governments and charities have been found to be significantly correlated with general mental health problems amongst refugees [12,18]. Insecure residency status in the displaced country or country of resettlement, also impacts mental health, due to uncertainty and fear about the future [12]. However, refugee women may experience unique issues with their mental and emotional health and wellbeing. These include post-natal depression, especially in those who have experienced trauma, vulnerability due to reliance on husbands, and gender-based violence and exploitation [19]. Women who have experienced interpersonal trauma such as assault may be more likely to develop posttraumatic disorder than men [20] and have been shown to have lower self-efficacy scores [18]. This may be due to their potentially lower social, linguistic and cultural integration in the resettlement country. Lower integration is likely to be a result of isolation due to child rearing responsibilities, which may preclude them from engaging in social activities such as employment [21,22,23]. Unskilled female migrants face particular challenges gaining employment due to a potential lack of education and formal qualifications, cultural expectations around family responsibilities and language barriers [24,25]. Social isolation and separation from family left behind contribute to anxiety, with opportunities for family reunion being limited by low socioeconomic status and harsh government policies [22,23]. The stress of transitioning from a homemaker to a provider may also be difficult [26]. Concerns about stigma from community members, distrust of authority figures or health professionals from different cultural backgrounds, and socioeconomic factors such as income, language and transport may be barriers to accessing appropriate mental health services [12,19].

### 1.2. Host Country Approaches toward Female Refugee Support

Resettlement policies fail to recognize the unique challenges faced by female refugees, with interventions focusing instead on males’ access to employment and financial independence [27]. More recently, a report by Kabir and Klugman [28] on the labor market and refugee employment across a variety of countries documented that refugee women may face administrative barriers and social discrimination even when they are legally employed.

For example, in Australia, female refugees experience higher rates of mental health issues and psychological distress than men [22,23]. In addition to social isolation from family members and limited opportunities for reunions, language barriers further contribute to this [29]. Ziersch et al. [22] offer anecdotal accounts of overt discrimination in a variety of settings, and of less obvious but implicit systemic institutional discrimination experienced in practices, policies or processes creating inequalities. For example, women from the Middle East in Australia experienced negative reactions arising from religious and cultural differences, and from distinctive and visible gender features such as the wearing of head coverings. Barriers and hardships which confront refugee women, often when compared with the more satisfactory settlement progress outcomes for refugee men are also noted in an Australian government review paper positing investment in refugees in Australia through a study of integration, employment and settlement outcomes [30]. Banulescu-Bogdan [31] recognizes and considers the challenges for refugee women seeking employment across Europe, North America and Australia such as language barriers, lack of in-demand skills, socio-cultural barriers such as child-care responsibilities and structural barriers such as restrictions caused by visa status. Banulescu-Bogdan [31] notes the lack of a coordinated response to this problem and proposes peer-to-peer relationships and mentoring as potential strategies for boosting social ties, economic empowerment and refugee integration.

### 1.3. Mentoring and Empowerment

Peer mentoring is usually undertaken by two or more people who are peers, with one acting as a mentor. Mentoring demonstrates a belief in the value of the individual and expresses a commitment to ongoing development, capacity building and enhancing agency [32]. Peer mentoring is a reciprocal process through which a more experienced individual encourages and assists a less experienced individual develop his or her potential within a shared area of interest. Peer mentors are individuals who share some common characteristics, attributes or circumstances such as age, ability, and interests; and who have more experience along with additional training in how to assist another in acquiring skills, knowledge and attitudes to be more successful [30].

Peer mentoring programs with marginalized populations integrate the principles of social justice, access and equity [33]. In the context of refugee/migrant CALD women undergoing cultural, linguistic and bureaucratic challenges in a host settlement country, the terms ‘mentors’ and ‘mentoring’ may be articulated, respectively, as ‘settled migrant’ and ‘empowerment support’. Sharing personal migratory narratives helps to build intimacy and connection within the group, promotes perceived social support among participants, and facilitates communal learning in a safe and relaxed environment [34,35]. The programs may lead to community inclusion, which in turn promotes a sense of belonging and improves health and wellbeing [36]. Peer support programs that provide an opportunity for participants to meet and share regularly have been shown to enhance quality of life, improve wellbeing and provide participants with strategies and confidence to overcome challenges and barriers. However, the literature on mentor(s) and mentoring is predominantly and historically focused on behavioral and organizational constructs in the primary and higher education, medical and business sectors [37,38]. Despite group programs and peer support models being utilized to support refugee communities in practice, very few of them have been evaluated with appropriate methodology [34]. The ability of peer mentoring programs to meet the specific needs of refugee and unskilled migrant women remain relatively unexplored [31].

Two main questions underpinned the review (1) “What are the impacts of community-based peer mentoring programs on the personal and employment outcomes for refugee and migrant women in resettlement countries?”, and (2) “What factors need to be considered in the design of a community-based participatory peer mentoring program to engage refugee and migrant women in resettlement countries?“.

Specific objectives were to: (i) describe peer mentoring programs that have been offered with refugee and migrant women in resettlement countries; (ii) identify social and wellbeing outcomes experienced by refugee women participants of peer mentoring programs; and (iii) identify key components of effective mentoring programs.

## 2. Materials and Methods

This systematic review was undertaken using standard methods according to the Preferred Reporting Items for Systematic Reviews and Meta-Analysis (PRISMA) [39]. Narrative synthesis was used to identify similarities and differences between studies and determine the strength of the evidence as it pertains to the review question [40].

### 2.1. Identification of Studies

Literature was sourced from the following electronic databases: ProQuest, Scopus, PubMed, Google Scholar and Wiley Online Library and included studies from 2005–2020. Reference lists of studies that matched the eligibility criteria were manually searched to identify further possible studies. Boolean operators and truncating of the distinct keywords in each search strategy combined with AND, OR and NOT were used to combine terms with each strategy and * was used for truncation where required.

The search terms used for:

1. The target population under review were CALD refugee and migrant (‘migrant’, ‘humanitarian migrant’, ‘refugee’, ‘asylum seeker’) women greater than 18 years of age;
2. The interventions and programs of interest were ‘peer mentor’/‘peer-led’/‘peer to peer mentoring programs’ targeted at CALD refugee and migrant women in resettlement countries with search terms related to the types of peer mentoring programs, ‘community based’, ‘participatory’, ‘structured’, ‘coaching’;
3. The outcomes were ‘resettlement’, ‘migrant support’, ‘peer mentoring process’, ‘integration’, ‘social inclusion’, ‘community support’.

Contextual and semantic text differences between studies required flexibility in the search process because certain words and phrases implicitly became substitutes for our original criteria keywords. For example, whereas ‘peer(s)’ may not initially surface in a keyword search, words such as, ‘friends’, ‘community’, and ‘support’ can tend to suggest some similarity in models of engagement with and among migrants and refugees.

### 2.2. Inclusion and Exclusion Criteria

Qualitative, quantitative and mixed-methods studies which presented the outcome/evaluation of peer mentoring/peer-led programs targeted at refugee and migrant CALD women, studies written in English, were considered in the review. Review articles, discussion papers, opinion papers, dissertations and theses, books, personal blogs, commentaries, articles from web pages, editorials, and articles written in languages other than English, were excluded. Despite an initial focus on peer mentoring programs for women only, studies that evaluated mixed gender refugee mentoring programs have been included in our evaluation if specific outcomes for women were reported. Studies were excluded if the mentoring programs were only for male participants, or where specific findings for female participants were not distinguishable. Mentoring programs for skilled migrants, typically located in the organizational context, were excluded as skilled migrants face different challenges to lesser-skilled refugees and migrants. A number of articles identified in the initial search provided overviews of general refugee/migrant support programs offered by community organizations to facilitate resettlement, some of which included aspects of peer support. However, if the programs did not include a specific mentoring program the articles were excluded.

**Figure 1:**
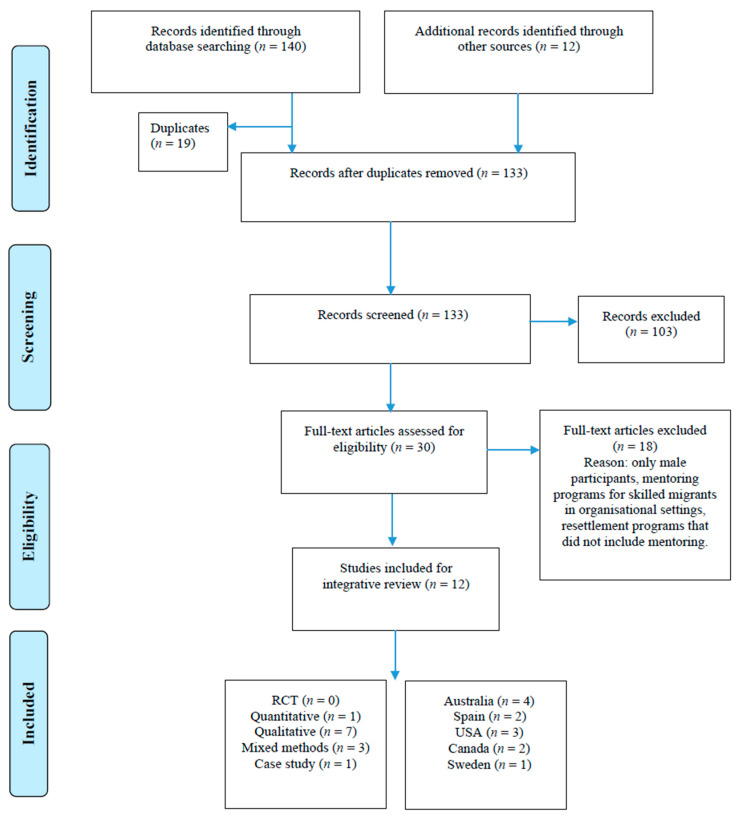
Literature selection process using PRISMA framework.

The initial search process identified 152 potential articles. Removal of duplicates resulted in the retention of 133 studies for abstract review to determine relevance and eligibility. Review of abstracts and titles led to the removal of a further 103. A total of 30 studies were retained for full review after 103 records were excluded. After full review, 18 were excluded as they did not meet the inclusion criteria. Figure 1 is the PRISMA flow diagram of the searching and screening of articles in this review. This process resulted in 12 articles for final inclusion in the review.

### 2.3. Quality Assessment of Articles

The Critical Appraisal Skills Program (CASP) checklists were used to assess each article [41]. Due to the lack of studies on this topic, and the acknowledged difficulty in reaching the refugee community [42], articles of a lower quality were accepted. Qualitative studies in this review were accepted even if data saturation was not achieved, or the researchers’ influence on the study was not addressed [43]. Mixed methods studies which did not describe the methods to combine data or analysis were also accepted [44]. The quantitative studies did not use randomization or a control group [45] but validated tools were used in data collection, which was considered acceptable.

### 2.4. Data Abstraction and Synthesis

The review objectives guided the data extraction process. The main characteristics that were extracted from the included articles were the country where the mentoring program was delivered, descriptions of mentees and mentors, key components of the mentoring program, methods of evaluation and outcomes (Table 1). The large number of qualitative studies, along with the variations in sample sizes and durations of the programs meant that thematic analysis was deemed the most suitable for synthesizing the findings [46].

## 3. Results

Of the 12 included studies (Table 1), all focused on refugee populations, 1 used a quantitative approach; 7 described qualitative studies, mostly using a participatory approach; 3 used a mixed methods approach and 1 was a case study. The mentoring programs were undertaken in Australia (*n* = 4, 33%), Spain (*n* = 2, 17%); the US (*n* = 3, 25%), Canada (*n* = 2, 17%) and Sweden (*n* = 1, 8%). Participant numbers in the included studies ranged from 6–172 [34,35]. Participants in the mentoring programs were Somali [48], Sudanese/South Sudanese [47,48,49], Burmese [47], Hmong, Afghani [47,50], Syrian [34], Bhutanese [51] or of non-specified mixed nationalities [35,52,53].

Five peer mentoring programs were delivered specifically to women and six were delivered to mixed gender groups. The remaining study focused on the mentors’ experience but provided information on key components of the program. As much as possible, only the outcomes for the female cohort of the program have been included in the review. All of the peer mentoring interventions were designed to improve refugee isolation and depleted social networks (all studies) and to improve host countries’ responses to the needs of resettled refugees [35,50]. Specific foci included mental health improvement (*n* = 4), social connectedness and social capital (*n* = 7) and employment (*n* = 1).

Descriptions of each of the mentoring programs, along with the main findings of the quantitative and qualitative evaluations, are outlined in Table 1. The key components of the programs are summarized below to show the commonalities in their structure and focus.

### 3.1. Key Components of Peer Mentoring Programs for Refugee Women

There were a number of common approaches to the development and composition of the peer mentoring programs. These are outlined below.

#### 3.1.1. Participatory Approach

In keeping with a participatory approach, the peer mentoring programs were commonly developed in collaboration with participants, via pre-intervention interviews or consultation [48,49]. This process ensured content was relevant to participant needs and was culturally appropriate [49]. In some cases, the mentors were heavily involved in the development of the program content, providing input on topics and content as they were simultaneously being trained to be peer mentors [51,53,54]. There was often a partnership between a community organization providing services for refugee communities and the university [35,50,53]. Staff of the community organizations and associated professional providers were also consulted in the development phase [53].

Community organizations were commonly the source of participant recruitment. Users of the programs and services offered by the community organizations were approached and invited to participate. When participants were recruited from the general community, without the support of a community organization, researchers had difficulty recruiting sufficient participants [52,53]. In the Swedish study, the employment mentoring program coordinators faced difficulties enrolling sufficient refugees in the program, and this resulted in a number of places in the mentoring program being filled by people who did not meet the criteria [55]. This highlighted the importance of working with community organizations who offer services directly to refugee populations.

In keeping with the principles of participatory research, the content of the programs evolved over time. Both mentees and mentors were consulted at various points to ensure the programs were meeting specific needs and content was added in response to ongoing feedback [51,53].

#### 3.1.2. Emphasis on Communication and Sharing

In accordance with the core principles of mentoring, the mentoring programs emphasized communication and sharing. Mutual exchange of knowledge was a key feature, either between mentors and mentees [50,51,52,53], or between mentees themselves [35,43,47,48]. Communication between mentors and mentees was verbal in all programs evaluated. The emphasis on verbal communication, rather than written, seems important, especially if verbal discussion is the typical medium of problem-solving in the culture of the refugees in the program [49].

There was a mixture of formats utilized by the mentoring programs, but were generally a combination of group workshops, and individual mentoring either face to face or by telephone. A total of 3 programs used workshops/group sharing only [34,51,53,54]; 1 used individual face to face mentoring only [52]; 2 programs used a mixture of group workshops and face to face individual mentoring [35,50]; 1 used a combination of group workshops and individual telephone mentoring [48] and 2 used telephone mentoring only [43,47,48]. The benefits of using alternative modes of engagement to suit different cultural preferences was noted [48]. The remaining study synthesized the results of a number of mentoring programs held in the community designed to help refugees find employment [55].

The workshop/group sharing format promoted socialization and allowed for the sharing of knowledge and the development of social capital between group members [35,48,50], particularly when workshops were held in the refugees’ language which increased acceptability [34,51]. Face to face contact and connecting with community members was valuable in building social networks and capacity. Guest speakers were utilized in several programs to provide specific education on particular issues [34,35,48]. However, limitations with the group approach in cultures where ‘preserving reputation’ is a cultural priority were noted by Stewart et al. [48] because participants felt reluctant to divulge problems (p. 26). Time management and scheduling were sometimes difficult as mentees sometimes worked many low-paying jobs (more relevant to males) or had competing priorities such as family commitments [48,51,52]. These factors, along with a lack of transportation options sometimes resulted in mentees arriving late to workshops and then wanting to revisit what had already been covered [48].

The individual mentoring format provided mentors with the flexibility to attend to individual participant needs [35,50]. This was particularly useful in participants with complex needs across several areas such as children’s access to education, health concerns and domestic violence support [35]. Where individual mentoring was offered in addition to group workshops, the individual mentoring allowed for a specific follow-up after the more generalized discussion in group settings [48]. However, where individual visits took place in the home, there was sometimes reluctance or suspicion shown by mentees’ families to accept the program [52].

Telephone mentoring was offered in several studies [47,48,49]. This took the form of either individual telephone support by mentors, or telephone access provided to mentees to enable them to support each other [47,48]. Refugee women may be restricted by geographical distance to their peers and mobile phones enabled that distance to be bridged [49]. Mobile phones were also a useful form of communication for stay-at-home mothers who may have lacked transport or had limited opportunities to leave the home.

#### 3.1.3. Accessibility

All mentoring programs took place in the community with workshops and group sessions being conducted at community centers, and individual home visits in some cases [35,52]. It was important that mentoring activities were undertaken in the communities where refugees were living, so that participants were not required to travel to universities or clinics [35,50]. A lack of transport is a known barrier in interventions that support refugees [48,51] and where travelling to community centers was difficult for participants, transport or telephone follow-up was provided [48]. One program was designed with the specific purpose of reaching house-bound women with no transport [52].

#### 3.1.4. Duration

There were conflicting findings on the optimum duration of the mentoring programs. Two mentoring programs lasted 8 weeks [51] and 10 weeks [49], respectively, and were deemed adequate in length [49]. However, other programs lasted 6 months and were considered both adequate [48,52] and inadequate [50] to cover all the complex issues that refugee women faced. These results indicate there is currently no definitive optimum length of a mentoring intervention for effective outcomes.

#### 3.1.5. Mentor Training and Support

Considerable focus was dedicated to the recruitment, training and support of mentors, and these were highlighted as essential components of the mentoring programs [35,48,50,52,53]. Mentors were recruited from a range of backgrounds. Four studies used mentors from the same language/cultural background as mentees [48,49,51,53], three used mentors from a range of nationalities, including Anglo/European English-speaking mentors [35,50,52]. Three studies reported that bilingual and bicultural mentors were effective during discussions of sensitive issues when cultural appropriateness was essential [35,48,52]. Having mentors with the same language background as the mentees enhanced the effectiveness of workshops and groups [48,51]. This was supported by Paloma et al. [53] and Badali et al. [34] who purposefully created language-specific groups for maximum effectiveness. However, in other mentoring programs where improving English language skills was one of the goals of the program, English-speaking mentors were effective [50,52].

The mentor training consisted mostly of responsibilities and roles, facilitation skills, strategies for assisting mentees with additional needs, the role of community services, and particular discussion themes [48,53]. The number of training hours ranged from 8 h [53] to approximately 48 h [50,53]. Training was sometimes shortened or interrupted due to the work and family commitments of the mentors [52,53]. One study described their mentoring training as ‘ongoing’, as there was no endpoint to their mentoring program. After initial training, mentors attended workshops with clients and received up-to-date information on a regular basis [35]. The remaining studies did not specify the length of training provided [34,48], stating only that it was undertaken by the research team, or not mentioning it at all.

Where the content of training programs was outlined, it contained a combination of communication skills, workshop facilitation skills, refugees’ emotional and practical needs, mentor responsibilities and understanding the migration experience [52,53]. For mentors with a different cultural background to the mentees, cross-cultural communication skills and specific cultural knowledge was important, along with practical advice on navigating home visits and accessing services [52]. With the exception of Paloma et al. [53], Im and Rosenberg [51] and Bond [52], little other description was provided of the content of mentor training sessions. However, it was noted that training materials and resource booklets were developed for mentors and made available for their use throughout the program [48,50,52,53]. On the completion of training, mentors were provided with supervision from the research team. In some cases, this was a series of structured weekly sessions [35,50], in others it was more ad-hoc [52]. Several authors stated the importance of ongoing supervision and support for mentors but noted this was a labor-intensive exercise [35,50,52]. Where support for mentors, and mentees, was not consistent, attrition occurred [52].

Consideration was given to the matching process where mentor/mentee dyads were formed. Dyads were matched according to ethnicity and gender [48], age, children and language [52], self-selected by mentees and mentors themselves after jointly participating in group sessions [50] or not mentioned [35,49]. It was noted that not all mentor/mentee relationships were sustained, and that matching could be unsuccessful, leading to attrition [52]. This was attributed to differences in expectations between mentors and mentees as to the goals of the program [52]. As a result of the sharing of knowledge and validation of their own journeys that occurred over the duration of the mentoring programs, mentors’ resilience and empowerment increased over the program [50,52,54].

#### 3.1.6. Cultural Considerations

There were a number of cultural considerations highlighted in the studies. It was considered important to have a culturally appropriate approach, particularly in the discussion of sensitive personal issues. Western-style counselling approaches with an individual focus were not considered appropriate [35]. Learning circles and group discussions suit participants from collectivist cultures [50,51], and refugee women from ‘oral’ traditions benefited from phone support or group support [48,49]. Interpreters were used when necessary to facilitate understanding [35,43].

The importance of the mentees’ families and communities accepting their participation in the mentoring program were highlighted, especially if this involved the mentor visiting the family home [43,52]. The consultation and involvement of community leaders assisted in this process [43]. Cultural expectations around the role of women that limited civic participation needed to be acknowledged and addressed [49,52]. Childcare needed to be considered, and where possible a creche was provided. Authors described the difficulties faced by women trying to take children to mentoring sessions by public transport, as refugee mothers did not always feel comfortable leaving their children with babysitters [48,52]. In this regard, home visits or telephone mentoring overcame this barrier [49,52].

Cultural issues sometimes resulted in attrition from the mentoring programs making it difficult for mentees to realize the full benefit [48,52]. Factors such as mismatched expectations between mentees and mentors [52], multiple dialects within groups making discussion difficult [48] and reluctance to speak in shy people and those wanting to save their reputation and not divulge personal issues were noted as barriers to group effectiveness [48].

Thematic analysis of the qualitative findings of the evaluations of the mentoring programs was undertaken to determine key outcomes [56]. Initially, eight codes were identified, and a coding framework was developed. The codes were condensed into categories, and in turn, themes. The themes related to obtaining support, reducing isolation and developing cultural understanding; building confidence and self-esteem; obtaining education and access to social services; and employment. These themes were condensed to four key outcomes for the women participants, being: Social support and connection, Wellbeing and personal growth outcomes, Improved access to the social determinants of health, and Employment outcomes.

### 3.2. Social Support and Connection Outcomes

Qualitative evaluations found that the mentoring programs resulted in improved access to social support and connection, including feeling less isolated and learning there are others in the same position [34,43,48,49,50,51,52]. Social networks were created, particularly as a result of the group sessions [34,43] and were noted as improving the community’s capacity to help each other [51]. However, having mobile phone numbers added another layer of connectivity between the participants, making it possible for them to connect between sessions [43,48]. In some cases, not only did the mentoring program enhance their social connectedness, but it also improved their social standing in the community, and within their families [49,51].

Attrition was noted in several studies, indicating that social connections were not always an outcome [52,53]. Authors attributed this to poor relationships between mentors and mentees, the competing priorities of mentees limiting involvement, and the high mobility of refugees as they move to seek employment [52,53].

### 3.3. Wellbeing and Personal Growth Outcomes

Two of the studies measured wellbeing outcomes quantitatively and found a significant positive impact on quality of life, distress, and post-traumatic growth [50,53]. The specific constructs of post-traumatic growth measured were appreciation of life, personal strength and relating to others. Whilst post-traumatic growth was measured in both the male and female mentees, scores were significantly improved in the female participants [53]. However, it should be noted that these results are from a small sample, without a control group, so results need to be interpreted with caution. There were no significant changes in scores for the final construct of ‘spiritual change’, however, the authors note this could be due to high baseline scores in this construct, or because it was not covered in the mentoring program. There were no significant changes in scores in happiness, or difficulty obtaining resources. Mentees continued to need support accessing resources after the completion of the mentoring program [50]. The improved quality of life scores were not maintained over the longer term, but remained above pre-intervention levels [50].

Empowerment was a noted outcome in several studies [43,50,53,54]. The mentoring process allowed for shared knowledge between mentees and mentors, validating the experiences of both, and leading to a sense of empowerment in both the mentors and the mentees. Whilst not measured empirically, participants reported in interviews their confidence and self-efficacy had improved through participation in the mentoring programs [49,50,52,54]. A greater capacity to cope both individually and as a community was noted [32,47]. Having a mobile telephone gave women confidence to leave their home to attend classes or appointments because they could still communicate with their children at home [43]. Mentees also described feeling they had more opportunities and could see new possibilities [53].

#### Improved Access to the Social Determinants of Health

Quantitative measures found increased scores post-mentoring in English proficiency, citizenship knowledge and satisfaction with resources [50]. These are areas that are essential for successful resettlement in host countries and improve access to the social determinants of health such as education. Participants were able to secure increases in resources through systems-based advocacy. Mentors helped mentees navigate various systems (education, health) to achieve goals and access resources and practical support for themselves and their families [48,49,50,52,53]. There was an increase in trust and understanding of community service providers such as the Police Service and Family and Children Services [34]. Improvements in health knowledge and skills were noted, along with subjective improvements in physical health [51]. However, it is not clear whether these results were sustained over time.

### 3.4. Employment Outcomes

There was little discussion of employment as an outcome for refugee women in the studies reviewed. Quantitative analysis of employment outcomes in Sweden for refugee participants in a mentoring program specific to enhancing employment noted no real impact for female refugee participants [55]. The authors conclude that being female reduces the chance of gaining employment, and that while mentoring may have some benefits for male refugees, no short-term benefit was identified for employment outcomes for female refugees [55]. Newman [57] noted that 12 months of active engagement with the refugee community in the UK resulted in some refugee women being employed in the community organization as volunteers, as students and as paid staff. However, the refugee women who ran the 12-month project did not have their employment extended [57]. Ongoing funding remained a barrier to the continuation of the project.

## 4. Discussion

This review sought to identify common core components of mentoring programs for refugee and migrant women in resettlement countries, and assess their effectiveness in improving wellbeing, social connection, and access to the social determinants of health such as employment. Results indicate that mentoring programs are effective in enhancing social connection and promoting wellbeing, but that these results are not necessarily sustained over time. Only one study measured the longer-term impact of participation in a mentoring program, and further research is needed to evaluate longitudinal impacts.

This review highlights that being able to participate in mutually supportive relationships is beneficial for refugee women. This may be with a mentor from the same language and/or culture, or with a locally born mentor, and benefits and disadvantages have been outlined for both approaches. There is value in providing opportunities for refugee women to share their stories with a mentor or group that practices empathic listening, prioritizes social connections and validates refugee women’s experiences [51,53].

Particular benefits were noted for using mentors with lived experience of migration and preferably forced migration [53]. Supportive peers can assist in helping refugee women overcome adversity and build on their known strengths and resilience [34,51]. This type of community peer intervention may be more effective than the utilization of professional care providers [53], or at least may facilitate a smoother transition to the professional health system. With regards to mental health in particular, Shishehgar et al. [58] posit that discussion groups for sharing refugee women experiences, while seeking social support from individuals who have endured similar experiences and consequential challenges may enable refugee women to ‘seek professional help in a timely manner’ for health problems (p. 960). However, the authors stop short of advocating for peer mentoring explicitly.

Building trusting relationships between organizations, communities and individuals takes time. In particular, refugee women are often a ‘seldom-heard’ group [57] and short-term approaches may not achieve meaningful results in terms of affecting successful resettlement [55,59]. Interventions need to be designed to allow sufficient time for each mentor/mentee interaction and to build social capital. Larger scale networking events between refugee communities and provider organizations may enable the development of relationships and trust with refugee communities, and an enhanced understanding of what is required for engagement [57,60]. Sulaiman-Hill and Thompson [42] highlight the importance of taking time to engage with participants as being critical to success. Providing community-centered programs enable refugee communities to take control of the community building process and strengthen ties both within the community and to the external community as well [51].

This review was constrained by the scarcity of relevant research outputs. Despite published refugee and migrant research spanning decades, it is apparent that peer mentoring of refugee women, and particularly, migrant women has not been well-examined. Furthermore, there are methodological limitations in the existing evaluations of mentoring programs. Very few studies are able to identify causality between specific components of programs and outcomes [61]. A clue to the reason for the paucity of research is offered by Sulaiman-Hill and Thompson [42] who refer to ‘a hidden population’ (p. 7) and how obtaining statistically representative samples of such groups is known to be problematic. Referring specifically to women participants, this article refers to females with limited education who are sometimes discounted in research studies or would often resist direct requests to participate. The authors note the challenges in overcoming indifference and wariness, with the best hope being the recruitment of enthusiastic people to employ snowball sampling. These trust and cooperation difficulties are borne out by Hynes [62] who purports that women’s experiences during displacement can lead to a lack of institutional trust.

There is little evidence that peer mentoring programs enhance employment outcomes for female refugees. This group may have gender-related limitations on labor force participation, such as family and caring responsibilities, language barriers, and limited work experience and training. Mentoring programs may be unable to address these [63]. Programs that build financial self-reliance and self-confidence in women, whilst acknowledging the time burden of child-care demands and the implicit employer preferences for hiring males are essential [63]. This aligns with a recent study of employment outcomes for Syrian refugees in Turkey, female refugees faced more barriers to employment due to gender biases in both the host and the source countries, expectations around the role of women in the home, lack of opportunities, sexual objectification and lower wages [64]. There remains an implicit, and sometimes overt, bias against the settlement and wellbeing interests of refugee and migrant women.

### 4.1. Building on Resilience

Many of the studies in this review concluded that outcomes for female refugee and migrant populations can be improved through better understanding of women’s values, perspectives, and expectations [48,49,50]. They encourage building on female refugees’ resilience and coping strategies to enhance settlement outcomes and wellbeing [51,65]. This aligns with previous literature on female refugee and migrants, which recommends using a strength-based approach in resettlement policies to achieve empowerment and meet refugees’ needs [66]. This may include religious strategies, which have been found to be a central component of coping with forced displacement [65].

Successful outcomes have also been achieved in health care research by building on women’s resilience and engaging with community networks to promote wellbeing [51,58,67].

### 4.2. Recommendations

Topic- and age-specific workshops may overcome confidentiality concerns for participants and enable facilitators to keep discussion to topics directly relevant to participants at different life stages. For example, workshops with a focus on employment or education issues [48]. Utilizing bicultural-bilingual mentors may enhance mental health and wellbeing outcomes. Western-style counselling roles should be replaced with more culturally appropriate activities such as psycho-educational information and case management [35]. Strong and consistent support is needed for mentors, with clear explanations of roles and flexible approaches to problem-solving. Longer-term funding is needed for community organizations to be capable of providing long-lasting programs that can create long-term change and to maintain community links [57].

There is scope for further research on this topic that could include: establishing the most effective content material for mentor training programs; and systematic and rigorous investigation of the effects of mentoring programs on participants’ wellbeing, self-reliance and social connectedness, especially over the longer term, using empirical methods suited to collectivist cultures’ definitions of wellbeing [54,68]. Further studies could also investigate the achievement of paid employment and attaining educational qualifications recognition, the cost-effectiveness of mentoring programs, and the value for money to host countries in improving economic integration [61].

## 5. Conclusions

This much needed and timely review provides valuable insights into the key components, challenges and contributing factors to successful outcomes in peer mentoring programs for refugee women. Mentoring programs can enhance female refugees’ wellbeing, build networks, improve interpersonal communication and social connectedness. However, the review highlights that for benefits to be sustained over the longer term, adequate support and continual funding is critical. The body of work in this area is currently limited and additional research is necessary to trial and rigorously evaluate other interventions to generate conclusive evidence for best practice.

## Figures and Tables

**Table 1:** Summary table–characteristics of studies included in review.

First Author, Year Country	Study Design	Mentees	Mentors	Intervention	Methods	Outcomes
Badali, 2017 [34] Canada	Qualitative study	11 refugees 6 women 5 men. Syria	Peer support. Participants mentored each other in the group.	12 weeks, 2.5 h once a week. Groups facilitated by Arabic speaker. Guest speakers. Gender-specific groups. Mental health concerns and settlement issues.	Focus groups (*n* = 2) One male group, one female group. In Arabic.	Improved community connection, social connections and support, development of a resilient mindset. Improved mental wellbeing through positive thinking and stress management. Improved self-care and self-worth. Positive impact on family wellbeing, demystification of community services. Built capacity for employment.
Khamphakdy-Brown, 2006 [35] USA	Case study	Refugee and immigrant women. 172 attended workshops. 17 had individual mentoring. Various countries.	Peer mentors: Bicultural-bilingual refugee or immigrant women Non-peer mentors: graduate students, university staff, community organization staff Numbers unavailable	Monthly. 2 h. Ongoing. Workshops—female refugee presenters Home visits/individual counselling-teams of 2 or 3. Professionals plus peers. Advocacy and case management. Mental health, acculturation/adjustment, physical health, family and gender roles, parenting, health, loss and grief, legal issues, unemployment and career barriers, and stress self-care counselling. To give women greater control over resources. 3 partners: University, community organization, domestic violence shelter.	Case study	Some evidence of a positive response to services. Numbers increased over time.
Liamputtong, 2016 [43] Australia	Qualitative study	Same sample as in Walker et al. [47]		Same program as in Walker et al. above Sub-objective: Further evaluation of the qualitative component of the foregoing described project.	Interviews (*n* = 29)	Creation of social networks, enhancement of wellbeing, reduction in isolation and provisioning of empowering experiences.
Stewart, 2012 [48] Canada	Qualitative study	27 refugee women 31 male refugees 39 Somalia 29 Sudan	11 Somali/Sudanese peer and professional, facilitators	12 weeks Design of a culturally congruent pilot test to meet support needs of two ethno-culturally distinct refugee groups. Groups/workshops. Bi-weekly; 60–90 min. 5–12 participants. Peer and professional facilitator. Information, affirmation and emotional support. Individual support via telephone. 20 min.	Interviews: mentees (*n* = 27) Mentors (*n* = 9) Field notes Focus group discussions (mentees) (*n* = 16 female)	Success in re-connecting people from African refugee communities; increased social integration; personal and practical support; decreased loneliness; expanded coping repertoire. Participants appreciated linguistic, gender and culture-specific grouping.
Wollersheim, 2013 [49] Australia	Qualitative study	9 Nuer (South Sudanese) refugee women	Peer support. Helped each other.	2 × 5-week periods; 20 h total. Pilot program, peer support. Participants were issued with mobile phone recharge vouchers to facilitate calls to each other. Small scale limited study designed to find out how to use mobile phone-based peer support to improve intragroup psychosocial health and to facilitate settlement.	2 Focus groups (both *n* = 9)	Increase in the existing and generation of new cognitive and social capital in the community lives, family lives, social lives and personal lives of participants. Greater confidence and empowerment. Verbal channel was effective, the form of communication they are most comfortable with. Program findings led to a further phase.
Walker, 2015 [47] Australia	Mixed methods study	111 refugee women 31 Afghan. 25 Burma (Buddhist) 11 Burma (Muslim) 44 Sudan	Peer support Helped each other	12 months Ongoing development of mobile phone-assisted peer support program discussed in Walker et al. (above) to support social connectedness among refugee women. Free-call use of mobile phones in culturally matched pairings.	Interviews (*n* = 29) Phone call logs Questionnaire constructed using measures from WHOQoL, Rosenberg’s Self-esteem Scale, the Efficacy Scale and the Friendship Scale.	Intervention provided communication pathways to improve interpersonal and social connections. Personal and practical support, and support in emergencies. Calls were primarily to peer group members, followed by nominated members of the heritage community. Fewest calls were made to the host culture service providers. The primary use of the phones, in all groups, was for peer support and a secondary use was for linkage with host society services. Questionnaire results not reported.
Goodkind, 2005 [50] USA	Mixed methods study	28 Hmong refugees 26 women 2 men	Undergraduate Students (*n* = 27) 19 European- Americans 8 migrant/peers	6 months, 6–8 h per week Community center in Hmong community Group learning circles- cultural exchange 1:1 support/advocacy English language, citizenship knowledge, accessing resources (education, healthcare etc.), self-efficacy. Systems-based advocacy. Strengths-based; Mutual learning; Validation through collective experiences	Quantitative: Basic English Skills Test (BEST) Immigration and Naturalization Services list of questions (used 10 out of 100) Satisfaction with Resources Scale Satisfaction with Life Areas Scale Rumbaut’s Psychological Well-Being Scale. Qualitative: Interviews	Significant positive impact on: English proficiency (*p* < 0.001); Citizenship knowledge (*p* < 0.05); Satisfaction with resources (*p* < 0.001); Quality of life (*p* < 0.05); Distress (*p* < 0.01) Most scores were not maintained after the intervention ended but remained above pre-intervention levels. No significant changes in happiness, or difficulty obtaining resources. Qual findings supported quant findings. Continue to need help accessing resources.
Im, 2016 [51] USA	Qualitative study	22 Bhutanese refugees 18 women 4 men	6 Peer mentors from the Bhutanese community. Mixed gender.	8 workshops over 2 months Wellness and healthy adaptation to resettlement. Mental and physical health focus, coping strategies.	Focus group discussions at the conclusion of each workshop.	Improvement in health knowledge and competency, better coping, building and strengthening social capital, sense of community and connectedness.
Bond, 2010 [52] Australia	Qualitative study	26 refugee women. Various countries. Average age 43 years. 16 withdrew	28 volunteer female mentors Mix of Anglo-Australian and migrant women 16 withdrew	Pilot project to provide personal and settlement mentoring to refugee women. 1:1 mentoring. Home visits and accompanying women to activities (shopping, medical, catching public transport, etc.) Engagement undertaken with broader community to recruit participants and mentors. Focus on agencies referring refugee participants.	Document analysis and interviews. Telephone Interviews: Coordinators (*n* = 3) Mentors (*n* = 8) Mentees (*n* = 7) Comm. Org. staff (*n* = 5)	The project was resource-intensive and difficult. However, progress was made toward model consolidation. Some improvement in social connectedness and confidence.
Paloma, 2020 [53] Spain	Mixed methods study	36 Refugees 20 men 16 women 17 countries	11 mixed gender mentors, mixed nationalities. Reduced to 6 after training.	15 weeks Community-based pilot intervention promoting posttraumatic growth (PTG) among refugee adults arriving in Seville. Phase 1—training of peer mentors to service the needs of refugees (8 weeks)Phase 2—culturally specific peer support groups Spanish speaking (*n* = 2) Ukrainian speaking (*n* = 1) French speaking (*n* = 1)	Post Traumatic Growth Inventory. Pre-post intervention (*n* = 27). Mentee written narratives Interviews Mentors (*n* = 5) mid-point and post-intervention	Significant post-intervention increases in PTG overall mean (*p* = 0.001); appreciation of life (*p* = 0.007); personal strength (*p* = 0.001); relating to others (*p* = 0.000). No significant difference for ‘spiritual change’. Degree of PTG improved significantly more in women than in men, and in middle aged participants, and those with university degrees. Deductive analysis of narratives showed findings aligned with PTGI sub-scales (above) but limited impact on spiritual change. Participants also described feeling they had more opportunities and could see new possibilities. Highlighted how PTG in the refugee population can be improved through a community-based intervention, specifically by adopting a peer-based mentorship approach
Paloma, 2020 [54] Spain	Qualitative study		Same cohort as previous Paloma et al. study	Analysis of mentors’ narratives was undertaken to explore processes of resilience and empowerment experienced by participants	Mentor journals and written feedback	Mentor resilience increased during first (training) phase, remaining high and stable for the second phase. Mentor empowerment steadily increased throughout.
Månsson, 2017 [55] Sweden	Quantitative study	122 male refugees Unspecified number of females Mixed nationalities	Nine community organizations. Mentors not culturally identified.	A variety of mentoring programs were run by the community organizations to facilitate employment. Study investigated the impact of the mentoring programs on the labor market statistics of newly arrived refugees. Metric data examination and questioning of the belief that mentoring is used as a mean to increase the speed of labor market integration of migrants. (Mixed gender data study).	Questionnaire pre-post Metric data from Employment Service database. Limited evaluation of female participants. Authors limited some of the analysis to males.	Being female reduces the chance of gaining employment. Completing the Swedish language course has a large positive effect on probability of employment (*p* < 0.05). Key finding is mentoring as a universal labor market ‘quick fix’ is unproven. Mentoring ‘shows promise’ for males. For females, no short-term effect is identified.

## Data Availability

Not applicable.
